# Nurse-Led Telephone Program for Nonadherent to Treatment Type 2 Diabetics With Comorbid Depression: A Cost-Consequence and Budget Impact Analysis

**DOI:** 10.1155/2024/9989080

**Published:** 2024-11-20

**Authors:** Manuel Gómez-Barrera, María Luisa Lozano-Del Hoyo, Juan Francisco Roy, María Teresa Fernández-Rodrigo, Piedad Gómez-Torres, Isabel Blázquez-Ornat, Sofía Pérez-Calahorra, Maria Esther Samaniego Diaz de Corcuera, Emilia Ferrer-López, Enrique Ramón-Arbués

**Affiliations:** ^1^Faculty of Health Sciences, San Jorge University, Villanueva de Gállego 50003, Zaragoza, Spain; ^2^Pharmacoeconomics and Outcomes Research Iberia, PORIB, Madrid, Spain; ^3^Department of Physiatry and Nursing, Faculty of Health Sciences, SAPIENF Research Group (B53-23R), University of Zaragoza, Zaragoza 50009, Spain; ^4^Las Fuentes Norte Health Center, Aragonese Health Service, Zaragoza, Spain; ^5^International University of La Rioja, Faculty of Health Sciences, Logroño 26006, Spain; ^6^University Institute of Environmental Sciences (IUCA), University of Zaragoza, Zaragoza, Spain; ^7^Research Group in Care (GIIS081), University Clinical Hospital Lozano Blesa, Institute for Health Research Aragón, Zaragoza 50009, Spain; ^8^SAPIENF Research Group (B53-23R), University of Zaragoza, Zaragoza 50009, Spain; ^9^Nursing Department, Nuestra Señora del Pilar Psychosocial Rehabilitation Center, Zaragoza, Spain; ^10^Nephrology and Renal Transplant Research Group-GIIS073, University Clinical Hospital Miguel Servet, Aragón Institute for Health Research IIS-Aragon, Zaragoza, Spain

**Keywords:** budget impact, cost-consequence, depression, tele-nursing, treatment adherence, Type 2 diabetes mellitus

## Abstract

**Objective:** To estimate the efficiency of a nurse-led telephone program for nonadherent to treatment Type 2 diabetics with comorbid depression (TELE-DD program).

**Design:** Secondary analysis of cost-consequence and budget impact, utilizing data from a randomized clinical trial conducted in the primary healthcare setting. The target population consisted of Type 2 diabetic patients with comorbid depression who were nonadherent to their pharmacological treatment.

**Method:** The average cost per controlled patient (glycated hemoglobin < 7%) and the incremental cost-effectiveness ratio were calculated. Similarly, the budgetary impact over 1 year of implementing this program in the region of reference of the randomized clinical trial was assessed.

**Results:** The number of controlled patients is higher in the TELE-DD group at 6, 12, and 18 months. The average cost per controlled patient was higher in the TELE-DD group than in the control group at 6 months (€160.31 vs. €49.79), but lower at 12 (€150.09 vs. €179.59) and 18 months (€209.22 vs. €376.88). The incremental cost-effectiveness ratio at 6, 12, and 18 months was €254.47, €143.65, and €177.46, respectively. The budget impact analysis revealed that implementing the TELE-DD program would result in a reduction of €721,940.68 in expenditure for the funder in the first year of application.

**Conclusions:** A nurse-led telephone program for nonadherent Type 2 diabetics with comorbid depression is an efficient option in the management of healthcare resources. These results highlight the role of nursing in chronic patient management and the efficient use of healthcare resources.

**Trial Registration:** ClinicalTrials.gov Identifier: NCT04097483.

**Summary**

• Implications for Practice and/or Patient Care: The implementation of a nurse-led psychoeducational telephone program such as TELE-DD is capable of improving metabolic control in patients with Type 2 diabetes mellitus (T2DM) and depression who are nonadherent to treatment, leading to cost savings for the healthcare system from 12 months of its implementation.
• Patient or Public Contribution: “No patient or public contribution.”
• Reporting Method: In this study, we adhered to relevant EQUATOR guidelines, and we followed the Consolidated Health Economic Evaluation Reporting Standards (CHEERS) reporting.

## 1. Introduction

Type 2 diabetes mellitus (T2DM) is one of the fastest growing noncommunicable diseases worldwide in recent years [1]. This disease and its associated complications have a significant economic impact [2, 3]. Global diabetes-related healthcare expenditures are estimated to have reached US $966 billion in 2021 and are projected to continue to increase in the future [4]. The enormous economic and social burdens resulting from T2DM demand new ways to improve disease control and, consequently, to curb healthcare spending.

## 2. Background

In this context, adherence to pharmacological treatment and lifestyle modifications (primarily healthy eating and physical activity) are key to achieving better economic and health outcomes [5]. It is estimated that in the United States, better adherence to diabetes medication could prevent many emergency department visits and hospitalizations each year, resulting in annual savings of up to $4.7 billion [6].

However, nonadherence to treatment for chronic diseases is a global problem that tends to increase [7], with the presence of depressive states being one of the strongest factors linked to this nonadherence [8, 9]. In the case of diabetes, the prevalence of depressive disorders is approximately twice the prevalence of depression in nondiabetics [10]. Evidence suggests that depressive symptoms and distress are associated with poorer self-care of diabetes and lower adherence to personal care regimens, such as glucose monitoring, diet, exercise, and poor medication compliance leading to worse glycemic control [11]. Therefore, this comorbidity ultimately results in a higher incidence of coronary heart disease, diabetes-related complications, hospital admissions, and ultimately higher healthcare costs [12]. Costs associated with depression and T2DM are high at an individual level, but comorbid diagnoses generate incremental costs greater than the sum of the costs of each diagnosis separately [13]. Thus, a recent study conducted in Germany found healthcare spending to be 42.2% higher in patients with T2DM with depression compared to those without depression [14].

From this perspective, the implementation of interventions specifically targeted at this patient population (T2DM + depression) that promote greater adherence to pharmacological and behavioral treatment for disease control is necessary. The TELE-DD program is a nurse-led telephone intervention that has promoted significant clinical improvements in this group of comorbid patients treated in the public health system of Aragon, Spain. The TELE-DD program employs a multicomponent approach that integrates educational, clinical, and emotional aspects, tailored to the individual needs of patients with T2DM and depression. This holistic integration is designed to enhance treatment adherence and clinical outcomes while addressing emotional well-being. TELE-DD offers a significant advantage over more rigid initiatives that do not consider these crucial factors for comprehensive and personalized care [15]. However, for the inclusion of new healthcare provisions, policymakers must consider not only clinical outcomes but also the costs associated with the implementation of new health strategies. The aim of this study was to analyze the efficiency of the TELE-DD program through a cost-consequence analysis and budget impact taking our regional public health system as reference.

## 3. Methods

### 3.1. Design and Setting

This study builds upon the results obtained from the TELE-DD clinical study, which evaluated the outcomes of implementing a nurse-led intervention for patients with T2DM and depression who were nonadherent to their pharmacological treatment. TELE-DD was an initiative developed within the primary healthcare setting under the auspices of the Health Planning Service of the Aragon Health Service. It was a psychoeducational intervention based on the guidelines outlined in the Joint Position Statement of the American Diabetes Association, the American Association of Diabetes Educators, and the Academy of Nutrition and Dietetics [16]. TELE-DD was framed within the cognitive-behavioral approach and was implemented through structured monthly telephone calls over 18 months, with an average duration of 20 min, conducted by nurses specialized in mental health and primary healthcare who were previously trained. The telephone contacts had a multicomponent, motivational, personalized, and adaptive approach.

The main objectives of the intervention were to promote treatment adherence, foster awareness of negative and/or erroneous thoughts, stimulate the physician–nurse–patient relationship, educate on healthy lifestyle behaviors, develop skills for managing emotional distress and problem-solving, and ultimately improve the quality of life and biopsychosocial well-being of patients. The protocol and the results related to effectiveness have previously been published.

### 3.2. Data Collection

Following the completion of this clinical trial, an economic analysis was conducted from the perspective of the public healthcare system of Aragon (Spain), as it is the financier of nursing services. Clinical and epidemiological data were extracted from the clinical trial, while the remaining necessary data, including economic quantification, were obtained from published literature. Additionally, all economic data extracted were updated to euros (€) of 2023 using the income updating tool from the National Statistics Institute (Instituto Nacional de Estadística) [17].

The reference clinical trial analyzed baseline data from 428 individuals (225 in the intervention group and 203 in the control group). In the intervention group, the mean age was 71.4 years (SD 10.3), the proportion of males was 28.4%, and the time since the diagnosis of T2DM was 10.2 years (SD 5.7). In the control group, the mean age was 71.5 years (SD 10.5), the proportion of males was 28.1%, and the time since the diagnosis of T2DM was 9.8 years (SD 5.5). No cases were statistically significant differences observed between the two groups. The outcome measure was the proportion of patients with T2DM controlled at 6, 12, and 18 months, accepting a value of HbA1c < 7% to define a patient with controlled T2DM. The results extracted from the TELE-DD trial are presented in Table 1.

### 3.3. Cost per Patient

In the present study, we followed the CHEERS reporting guidelines [18]. For the estimation of the costs of implementing this intervention, we assumed 120 min per semester per nurse and patient, in addition to a routine quarterly visit. In the control group, the same routine quarterly visit was assumed. The duration of a nursing consultation was estimated at 10 min [19], and the cost per hour of a primary care nurse was estimated at €17.35 based on a payroll including base salary plus experience supplements in our reference healthcare system. The cost per patient is presented in Table 2.

### 3.4. Results Calculated

Due to the outcome variables used corresponding to units of clinical outcome, a cost-consequence analysis was conducted. The time horizon used was 6, 12, and 18 months as these were the outcome assessment time points from the original clinical trial; the discount rate was not considered as there were no interannual comparisons. The average cost per controlled patient (MCER) corresponding to the mean cost per controlled T2DM patient was calculated, as well as the incremental cost-effectiveness ratio (ICER) corresponding to the additional cost required to achieve an additional outcome with the implementation of TELE-DD. A univariate sensitivity analysis was performed based on the 95% confidence interval (CI) of the clinical outcomes obtained to analyze the robustness of the results in the least and most favorable scenarios. MCER and ICER were recalculated according to CI limits of patients with HbA1c < 7% at 6, 12, and 18 months. Minimum value of CI was considered less favorable scenario, and maximum value of CI was considered more favorable scenario. If the main conclusions are maintained in different scenarios and base case, the robustness of the results will be achieved.

Similarly, the budget impact of implementing this program in the Autonomous Community of Aragon for the group of nonadherent patients with T2DM and depression was calculated. The time horizon used was 1 year, so no discount rate was applied. To estimate this budget impact, it was assumed that the hiring of three nurses responsible for coordinating and managing the implementation of the program would be necessary.  Table 3 presents the parameters considered in the budget impact model, as well as their sources.

### 3.5. Ethical Considerations

The present economic analysis is based on the clinical results described in the TELE-DD clinical trial [20]. The TELE-DD project was previously evaluated and approved by the Health Research Commission Sector II of HEALTH, Secretary of the Unit of Healthcare Quality and Assistance of HEALTH, authorized by the Clinical Research Ethics Committee of Aragon, Spain (CEICA, Exp. CP-CI-PI17-0167), and registered in ClinicalTrials.gov. TELE-DD was designed following the ethical standards outlined in the Declaration of Helsinki and its subsequent amendments.

## 4. Results

Between May 2018 and February 2020, 428 participants were randomized. The TELE-DD group (*n* = 225) and the usual care group (*n* = 203) were similar at baseline [22].

### 4.1. Cost-Effectiveness

**Figure 1:**
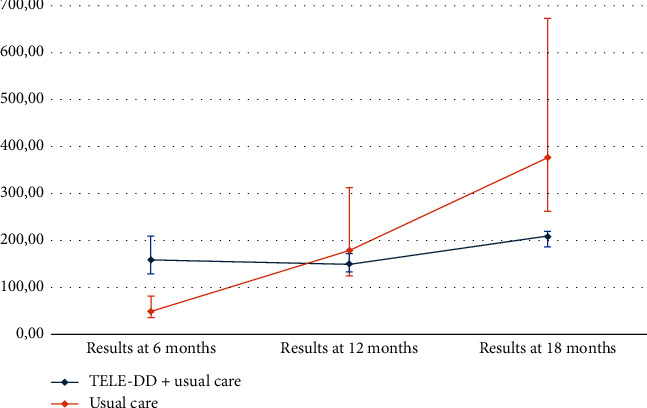
Mean cost per controlled T2DM patient (CI 95%) at 6, 12, and 18 months (€).

**Figure 2:**
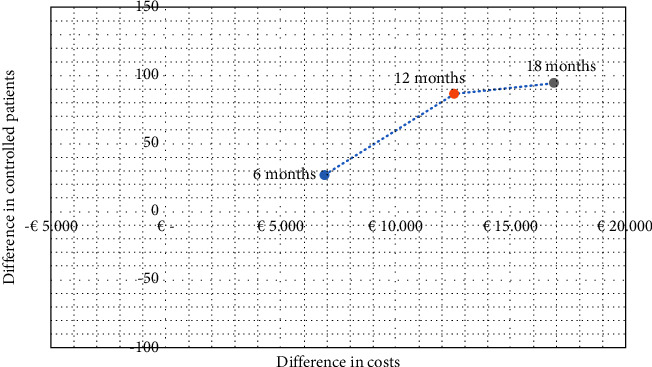
ICER in 6, 12, and 18 months in cost-effectiveness plane.

Table 4 presents the results of MCER and ICER cost per patient with controlled DM. It can be observed that the number of controlled patients is higher in the TELE-DD group at 6, 12, and 18 months, and the costs are also higher. The MCER corresponding to the cost of obtaining a controlled patient is higher in the TELE-DD group at 6 months, €160.31 compared to €49.79. However, at 12 and 18 months, this MCER is lower in the TELE-DD group, €150.09 compared to €179.59 at 12 months and €209.22 compared to €376.88 at 18 months (Figure 1). This means that at 12 and 18 months of implementing this intervention, the investment required to achieve a controlled patient is reduced, and the use of the intervention to ensure that a nonadherent patient with T2DM and depression becomes controlled is more efficient.  Figure 2 shows the cost-effectiveness plane with the evolution of the ICER at 6, 12, and 18 months. The shift of the points upward and to the right implies that the increase in costs is associated with an increase in results, which is more noticeable in the short term when a greater number of patients are monitored. The sensitivity analysis showed the best results for TELE-DD in the more favorable scenario with respect to a less favorable scenario because the difference between the two alternatives is higher in the upper limits of the CIs of controlled patients. In the most favorable scenario at 12 months, the cost per controlled patient is slightly higher (€7.15) in TELE-DD but is fairly lower in the base case and in a less favorable scenario and in 18 months; we can conclude that the higher benefit of the TELE-DD program will be reached between 12 and 18 months.

### 4.2. Budget Impact

Table 5 presents the results for the target population for the implementation of the TELE-DD intervention across the entire autonomous community of Aragon.  Table 6 presents the results of the program implementation; it can be observed that the implementation of TELE-DD in patients with T2DM and nonadherent depression would result in a reduction in expenditure of €721,940.68. This difference is attributed to the higher number of patients with controlled T2DM, 1729 in TELE-DD compared to 311 in standard treatment, and their lower cost.

## 5. Discussion

Previously, we demonstrated that the implementation of a telephonic psychoeducational program led by nursing professionals, such as TELE-DD, improved metabolic control in nonadherent patients with T2DM and depression [22]. In this study, we have shown that a moderate investment like this intervention can lead to savings for the healthcare system starting from 12 months of implementation.

Within the first year, TELE-DD participants showed a much higher probability of achieving HbA1c levels < 7% compared to patients under usual care (54.3% vs. 9.8%). These clinical results were achieved with a lower MCER (€150.09 vs. €179.59 for usual care) and a very reasonable ICER (95% CI) of 143.65 (153.38–135.08) per controlled patient (HbA1c < 7%) at 12 months. At 12 months, the sensitivity analysis in a more favorable scenario showed a slightly higher cost (€7.15) in the TELE-DD group, indicating that, in patients with a potentially more favorable response, the intervention may require more time to demonstrate its efficiency.

The resulting budget impact analysis suggests that the TELE-DD program could lead to a significant cost reduction for the public healthcare system (−€721,940.68 per year of implementation).

We believe that the strength of the TELE-DD program lies in two fundamental aspects: (i) its nursing leadership, unlike usual care where family physicians are more actively involved, and (ii) its cognitive-behavioral approach, which contrasts with the predominantly informative and prescriptive model of usual care.

The clinical and economic importance of the nursing role in managing chronic processes such as T2DM has been previously reported. In economic terms, [23] observed a lower likelihood of hospitalization and emergency service use in diabetic patients attended by nurses compared to those attended by doctors, resulting in cost savings of $500 per patient per year. Similar trends have been observed in the management of different profiles of chronic patients [24, 25].

Behavioral change strategies, similar to the intervention analyzed in this study, have shown economically positive results, both from the healthcare system and societal perspectives [26]. A recent systematic review by the National Institute for Health Research [27] concluded that intensive interventions based on a cognitive-behavioral approach are more cost-effective than those based solely on counseling or usual care. This finding may be even more relevant in people with depression, such as the target population of TELE-DD. People with depressive disorders tend to be less adherent to T2DM treatment [28], and in turn, they may benefit more from interventions with a cognitive-behavioral approach, which is a preferred psychological care paradigm in managing depression. In this regard, recent systematic reviews with meta-analyses conclude that cognitive-behavioral interventions improve depressive symptoms in people with chronic diseases overall [29, 30], including diabetes in particular [31]. From this perspective, it is plausible to assume that such interventions can enhance psychological health, self-control behaviors, subsequent medical outcomes, and ultimately, the costs associated with patients with T2DM and depressive disorder.

Currently, several factors need to be considered in healthcare budget allocation strategies. The severity and magnitude of the disease, budgetary impact, availability of treatment alternatives, and legal, ethical, and organizational issues play a fundamental role in decision-making [32]. However, healthcare is a field where needs grow at a faster rate than resources to meet them. Therefore, policymakers need more economic studies to aid decision-making. Currently, patient self-care support programs are structurally underfunded, and healthcare economic research on these strategies is underrepresented compared to evaluations of healthcare technologies and medications [33]. To our knowledge, this is the first study to analyze the balance between cost and outcomes of a nurse-led telephone intervention for managing T2DM in patients with depression. Our results indicate that it is a useful and cost-effective strategy for managing these patients and an effective cost-saving strategy for the healthcare system. To maximize the benefits of this initiative and ensure its long-term sustainability, several key policies should be implemented. Including the TELE-DD program as an essential component of public primary care services is crucial to ensure that all patients with T2DM and depression have access to this intervention. Establishing ongoing training plans for nurses in motivational interviewing and collaborative care techniques is imperative. Keeping staff up-to-date will enhance the program's effectiveness and increase patient satisfaction. Additionally, a robust monitoring and evaluation system should be developed to measure the program's effectiveness and make adjustments based on empirical data. This system will help identify areas for improvement and ensure that the program remains aligned with its objectives. Implementing these recommendations can optimize the TELE-DD program, ensuring its long-term effectiveness and sustainability, and providing a replicable model for other public health interventions. Policymakers should account for these funding needs, considering the potential medium- and long-term savings in hospitalization and emergency care costs.

Some limitations of this study should be noted. We opted for a perspective of direct costs from the healthcare resource funder instead of a broader social perspective; future analyses specifically oriented would provide more information on relevant costs derived from productivity losses of younger patients and formal or informal caregivers of older patients. In this regard, it is estimated that in Spain, the direct costs associated with T2DM amount to €5.1 billion, and the costs associated with productivity loss amount to €2.8 billion [34]. Additionally, our analysis only provides economic results related to the control of T2DM; it does not calculate the savings associated with the improvement in mental health or other clinical parameters (such as LDL cholesterol and blood pressure values) achieved by the TELE-DD [20]. It is reasonable to assume that by including these parameters, the benefit derived from the implementation of the TELE-DD program could be even higher than reported here.

In addition, our analysis only provides economic results related to T2DM control and does not calculate the savings associated with improvements in mental health or other clinical parameters (LDL cholesterol and blood pressure values) achieved by TELE-DD. It is reasonable to think that including these parameters, the benefit derived from the TELE-DD program implementation may be even higher than reported here. Lastly, this study only reports the economic results of 18 months of intervention. At this point, two factors suggest the need for further analysis over longer time horizons. The implications of long-term research on the TELE-DD program are significant and multifaceted, addressing both patient response and severe complications associated with poor control of T2DM. First, understanding the long-term response of patients to such initiative is crucial for assessing the sustainability and continued effectiveness of the intervention. This research would enable the identification of whether the benefits observed in the first 18 months are maintained, improved, or diminished over time. Additionally, it could reveal long-term patterns of adherence and behavior, providing valuable data on how patients integrate self-management strategies into their daily lives. These findings could inform adjustments and improvements to the programs, ensuring their relevance and effectiveness over the years. Second, severe complications from poor control of T2DM, such as cardiovascular disease, neuropathy, nephropathy, and retinopathy, may develop over the years and significantly impact the economic outcomes of the initiative. Long-term research would allow for an assessment of how such an intervention influences the incidence and progression of these complications. If the initiative proves effective in reducing these complications, the economic savings could be substantial, not only in terms of direct healthcare costs but also in reducing the need for hospitalizations and emergency treatments. Furthermore, a lower incidence of severe complications would improve the quality of life for patients, potentially translating into increased productivity and lower costs associated with lost productivity and informal care. These findings would be critical to justify continued investment in the program and could serve as a basis for its expansion and adaptation to other populations and contexts.

## 6. Conclusion

The findings of this economic study suggest that in patients with T2DM and depression, a multicomponent, motivational, and personalized telephone intervention such as that proposed in the TELE-DD program improves T2DM control and is an efficient option in managing healthcare resources. These results underscore the importance of promoting self-care support strategies in chronic patients and highlighting the fundamental role of nursing in controlling chronic patients and efficiently using healthcare resources.

## Figures and Tables

**Table 1:** Results of the TELE-DD program.

Patients with HbA1c < 7%	TELE-DD + usual care *n*; % (IC 95%)	Usual care *n*; % (IC 95%)
6 months	50; 25.3 (19.2–31.3)	23; 11.6 (7.2–16.1)
12 months	106; 54.4 (47.4–61.4)	19; 9.8 (5.6–14.0)
18 months	113; 59.2 (52.2–66.1)	18; 9.4 (5.3–13.6)

*Note:* Percentage of patients with HbA1c < 7%.

Abbreviations: CI, confidence interval; HbA1c, glycated hemoglobin type A1c.

**Table 2:** The cost per patient. Intervention and control group cost.

	TELE-DD + usual care	Usual care
Minutes of intervention per semester	120	—
Routine care per semester	20	20
Cost per patient at 6 months (€)	40.48	5.78
Cost per patient at 12 months (€)	80.97	11.57
Cost per patient at 18 months (€)	121.45	17.35

Abbreviation: €, euro.

**Table 3:** Parameters considered in the budget impact model.

Parameter	Value	Source
Population of Aragon over 18 years old	1.107.072	National Statistics Institute (INE) [17]
Target population (T2DM + depression + nonadherence)	0.333%	Roy et al., 2021 [20]
Cost per patient with T2DM∗ Controlled Uncontrolled	€3.628.56 €4.224.02	Mata-Cases et al., 2016 [21]

Abbreviation: T2DM, Type 2 diabetes mellitus.

^∗^Prices updated to € of 2023 (National Statistics Institute (INE) of Spain, n.d.).

**Table 4:** MCER and ICER cost per patient. Cost- effectiveness results.

Results at 6 months	TELE-DD + usual care (*n* = 198)	Usual care (*n* = 198)
Costs (€)	8.015,70	1.145,10
No. of controlled patients (CI 95%)	50 (38–62)	23 (14–32)
MCER, base case (less favorable–more favorable) (€)	160.31 (210.84–129.32)	49.79 (80.85–35.97)
ICER base case (less favorable–more favorable) (€)	254.47 (288.02–227.92)	
Results at 12 months	TELE-DD + usual care (*n* = 195)	Usual care (*n* = 194)
Costs (€)	15.909,95	3.412,17
No. of controlled patients (CI 95%)	106 (92–120)	19 (11–27)
MCER, base case (less favorable–more favorable) (€)	150.09 (172.25–132.99)	179.59 (313.45–125.84)
ICER base case (less favorable–more favorable) (€)	143.65 (153.38–135.08)	
Results at 18 months	TELE-DD + usual care (*n* = 191)	Usual care (*n* = 191)
Costs (€)	23.642,27	6.783,85
No. of controlled patients (CI 95%)	113 (100–126)	18 (10–26)
MCER, base case (less favorable–more favorable) (€)	209.22 (213.17–187.17)	376.88 (672.60–261.78)
ICER base case (less favorable–more favorable) (€)	177.46 (188.15–167.91)	

Abbreviations: CI, confidence interval; ICER, incremental cost-effectiveness ratio; MCER, mean cost-effectiveness ratio.

**Table 5:** Target population for budget impact calculation.

Description	Data	People
Population of Aragon over 18 years old (National Statistics Institute, n.d.)		1.107.072
% Target population (T2DM + depression + nonadherence) [20]	0.333%	3.690
% Poorly controlled patients [20]	86.2%	
Poorly controlled DM patients, baseline		3.181
Well-controlled DM patients, baseline		509

Abbreviation: %, percentage.

**Table 6:** Budget impact of TELE-DD implementation due to T2DM control.

Description	TELE-DD + usual care	Usual care
% of patients controlled per year	54.4%	9.8%
Patients controlled per year	1.729	311
Patients not controlled per year	1.451	2.869
Cost of controlled patients (€)	6.273.780,24	1.128.482,16
Cost of noncontrolled patients (€)	6.129.053,02	12.118.713,38
Three TELE-DD coordinating nurses (€)	122.421,60	—
Total cost (€)	12.525.254,86	13.247.195,54
TELE-DD-standard treatment difference (€)	−721.940.68	

*Note:* Negative values indicate cost savings with TELE-DD compared to standard treatment.

Abbreviation: €, euro.

## Data Availability

The data that support the findings of the study are available from the corresponding author upon request.
